# Fixation of delayed distal radial fracture involving metaphyseal diaphyseal junction in adolescents: a comparative study of crossed Kirschner-wiring and non-bridging external fixator

**DOI:** 10.1186/s12891-020-03404-0

**Published:** 2020-06-09

**Authors:** Jin Li, Saroj Rai, Xin Tang, Renhao Ze, Ruikang Liu, Pan Hong

**Affiliations:** 1 Department of Orthopaedic Surgery, Union Hospital, Tongji Medical College, Huazhong University of Science and Technology, Wuhan, 430022 China; 2 Department of Orthopaedics and Trauma Surgery, National Trauma Center, National Academy of Medical Sciences, Kathmandu, Nepal; 3 First School of Clinical Medicine, Tongji Medical College, Huazhong University of Science and Technology, Wuhan, China

**Keywords:** Distal radius fracture, Metaphyseal diaphyseal junction, External fixator, Crossed Kirschner-wiring

## Abstract

**Background:**

Conservative treatment remains the preferred choice for distal radius fracture in children. However, loss of reduction is problematic, especially in an older child. Crossed Kirschner-wires is widely used to treat distal radius fracture in adolescents. This study aimed to compare the application of crossed Kirschner-wiring (KW) and non-bridging external fixator (EF) for the treatment of delayed distal radial fracture involving metaphyseal diaphyseal junction (MDJ) in adolescents.

**Methods:**

Between January 2012 to January 2017, 146 (male = 101, female = 45) patients in EF group and 117 (male = 76, female = 41) in KW group, were reviewed retrospectively. Preoperative data were collected from the hospital database, and postoperative clinical outcomes data were collected during the follow-up visits. We used SPSS for data analysis.

**Results:**

There existed no significant difference between EF and KW regarding sex, body weight, fracture side, duration from injury to surgery. The duration of surgery was significantly shorter in EF (30.5 ± 6.1 min) than the KW group (44.6 ± 9.4 min), *P* < 0.001. The number of intraoperative X-ray images was significantly lower in EF (6.5 ± 1.1) than KW (11.8 ± 2.3), *P* < 0.001. The incidence of tendon irritation is significantly higher in the KW (19.7%) than the EF group (0%), *P* < 0.001. The residual angulation on the AP view was higher in KW (3.8 ± 2.3, degrees) than the EF group (2.5 ± 1.6, degrees), *P* < 0.001. The volar tilting is better in EF (6.6 ± 1.1, degrees) than the KW group (1.0 ± 1.5, degrees), *P* < 0.001. However, the functional outcomes of the wrist showed no significant difference between EF and KW group, *P* = 0.086.

**Conclusion:**

The EF was superior to KW in the treatment of radial MDJ fractures in adolescents. The EF displayed shorter duration of surgery, less tendon irritation, and better radiographic outcomes than the KW. However, the cost-effect analysis remains to be investigated, because the EF is more expensive than KW.

## Background

**Fig. 1:**
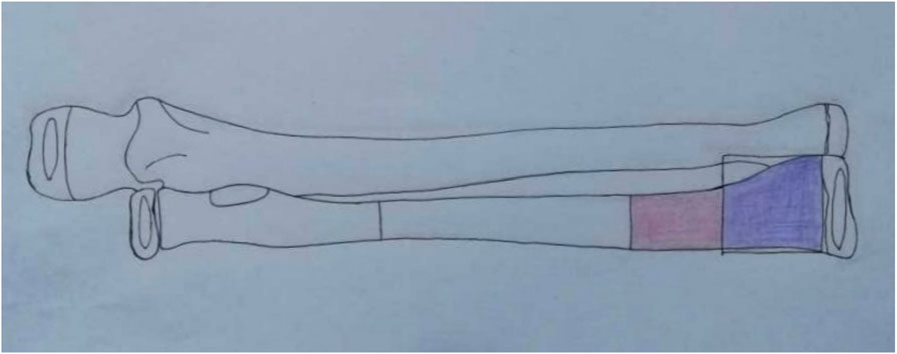
Schematic illustration of the fracture in this study (anteroposterior view). The blue square is a square area whose side has the same length of the distal physis of the radius; We defined the MDJ (red area) as the distal third of radius subtract the blue square

Distal radius fracture is a common injury in children and adolescents [1]. The nonoperative method of treatment remains the primary choice for the pediatric population, especially in younger children [2, 3]. Adolescents with similar injury patterns of the distal radius as in adults display a unique fracture, and the vulnerable zone is usually the metaphyseal diaphyseal junction (MDJ) [4]. We defined the MDJ as the distal third of radius subtract the square over the radial physis (Fig. 1), and it is a reproducible basis for evaluation.

Because of limited remodeling potential and higher demand for wrist function in adolescents, surgical intervention was advocated [5]. The surgical intervention includes plates [6], elastic stable intramedullary nail (ESIN) [7, 8], and Kirschner wire (KW) [4, 9]. It is indicated if (1) the fracture is unstable or difficult to reduce, (2) loss of fracture reduction at follow-up radiographs: more than 15 degrees on sagittal plane, more than 5 degrees on frontal plane, and (3) impending compartment syndrome with the cast.

Surgery is meant for better stability and accurate fixation. However, all these techniques have various disadvantages of being technically demanding, and plating requires a large incision and is usually unacceptable to the patients and parents.

As a tertiary medical center, the majority of patients at the out-patient visit were transferred from local hospitals. Delayed presentation of displaced distal radius fractures in adolescents was common as manual reduction and casting or splinting were usually tried for most of the patients before arrival at our hospital. We considered a delayed presentation if the patients arrive hospital after 72 h. Antegrade ESIN was quite challenging and is not a preferred option in our hospital. Before the introduction of an external fixator (EF), KW was our preferred choice. A simple technique using EF to stabilize MDJ fractures was introduced in our institute since 2012. It is a simple and minimally invasive surgical procedure and has a short learning curve.

This retrospective study aims to compare the outcomes and complications of EF and crossed KW for the treatment of delayed MDJ fractures in adolescents.

## Methods

### Study population

All patients in this study were treated surgically in the Department of Orthopaedic Surgery, Wuhan Union Hospital, Tongji Medical College, Huazhong University of Science and Technology, between January 2012 and January 2017. Patients were categorized into the EF group and the KW group according to the surgical procedures.

Patients in this study were required to fit following criteria: (1) 10–14 year-old; (2) complete fracture of the distal radius, with or without ulna fracture; (3) failed trials of nonoperative treatment; (4) two-part fracture without comminution or with slight comminution; (5) injury to surgery more than 3 days; (6) MDJ fractures with open visible physis on the radiographs.

Exclusion criteria were as follows: (1) severely comminuted fractures; (2) radial styloid fractures; (3) fractures associated with neurovascular injuries; (4) pathological fractures or open fractures; (4) duration from injury to surgery over 14 days.

Baseline information including sex, age, operative side, absence/presence of ulnar fracture, duration from injury to surgery, duration of operation, number of intraoperative X-ray images, duration from surgery to hardware removal, rotation of forearm, were reviewed retrospectively from the hospital database. The functional outcome of the wrist was measured at the follow-up visit, using the Disabilities of the Arm, Shoulder and Hand (DASH) Score [10]. Fractures union was evaluated by radiographs, including wrist joint and full-length forearm, at every out-patient visit.

### Surgical technique

The surgery was performed under brachial plexus block (BPB) or general anesthesia (GA), without the usage of the pneumatic tourniquet. The forearm was placed on a radiolucent table, and the surgery was performed under fluoroscopic guidance.

**Fig. 2:**
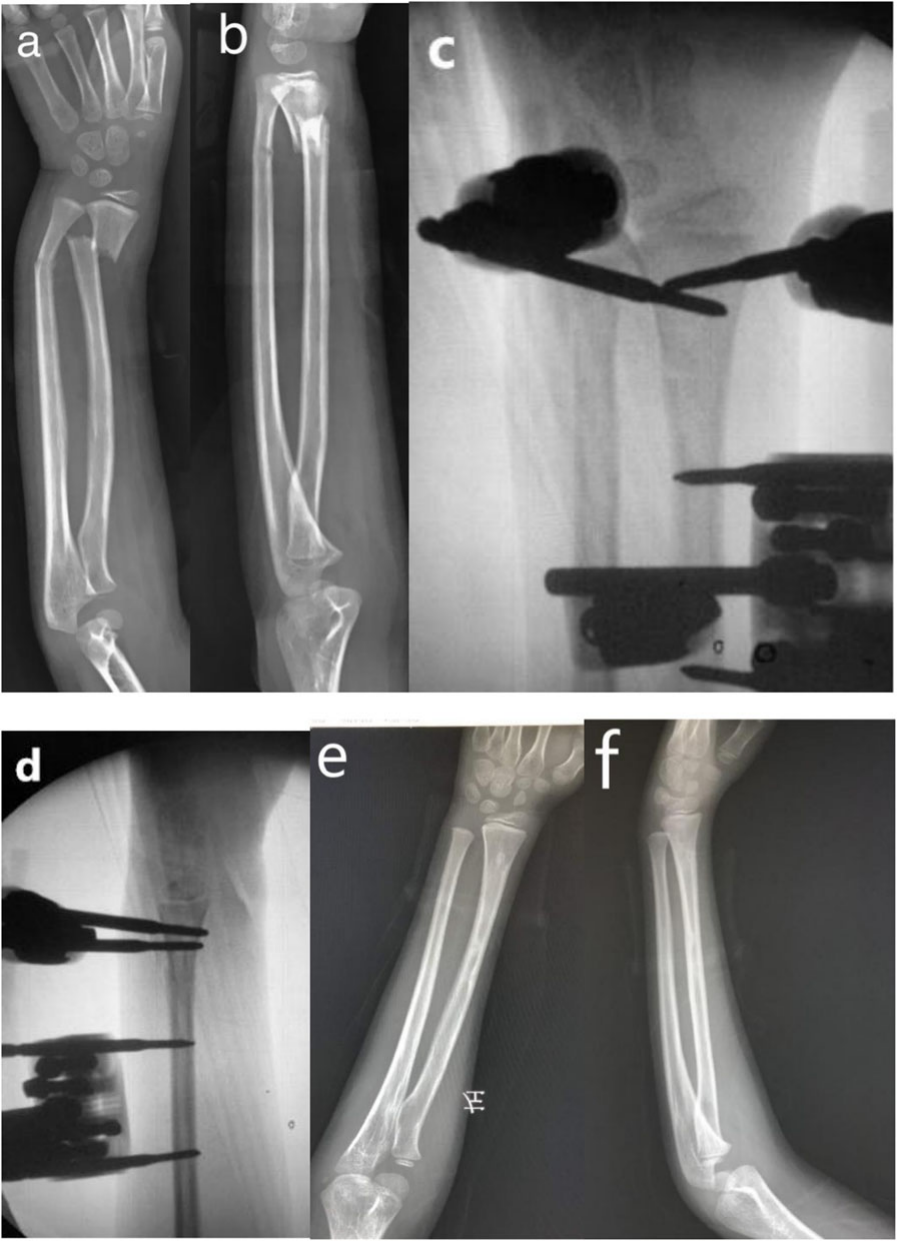
11-year-old boy with left distal radius-ulna fracture treated with external fixator. **a** AP view of forearm before surgery. **b** Lateral view of forearm before surgery. **c** AP view of forearm after surgery. **d** Lateral view of forearm after surgery. **e** AP view of forearm at 12th month follow-up after surgery. f. Lateral view of forearm at 12th month follow-up after surgery

The external fixator application was performed as follows. The fracture was manually reduced until the satisfactory alignment was achieved, and two Schanz pins (Tianjin Xinzhong Medical Devices Co. Ltd., Tianjin City, China) were placed in the distal fracture fragment. The insertion point of the distal-most screw should be at least 5–10 mm proximal to the physeal line. Then, the other two screws were placed in the proximal fracture fragment radially, and the screws were connected with proper clamps and rods. The fracture alignment was confirmed with fluoroscopy on anterior-posterior (AP) and lateral view. Sometimes, if the manual reduction was difficult, a K-wire was utilized as a lever to facilitate the reduction. Usually, the ulna was not fixated (Fig. 2).

**Fig. 3:**
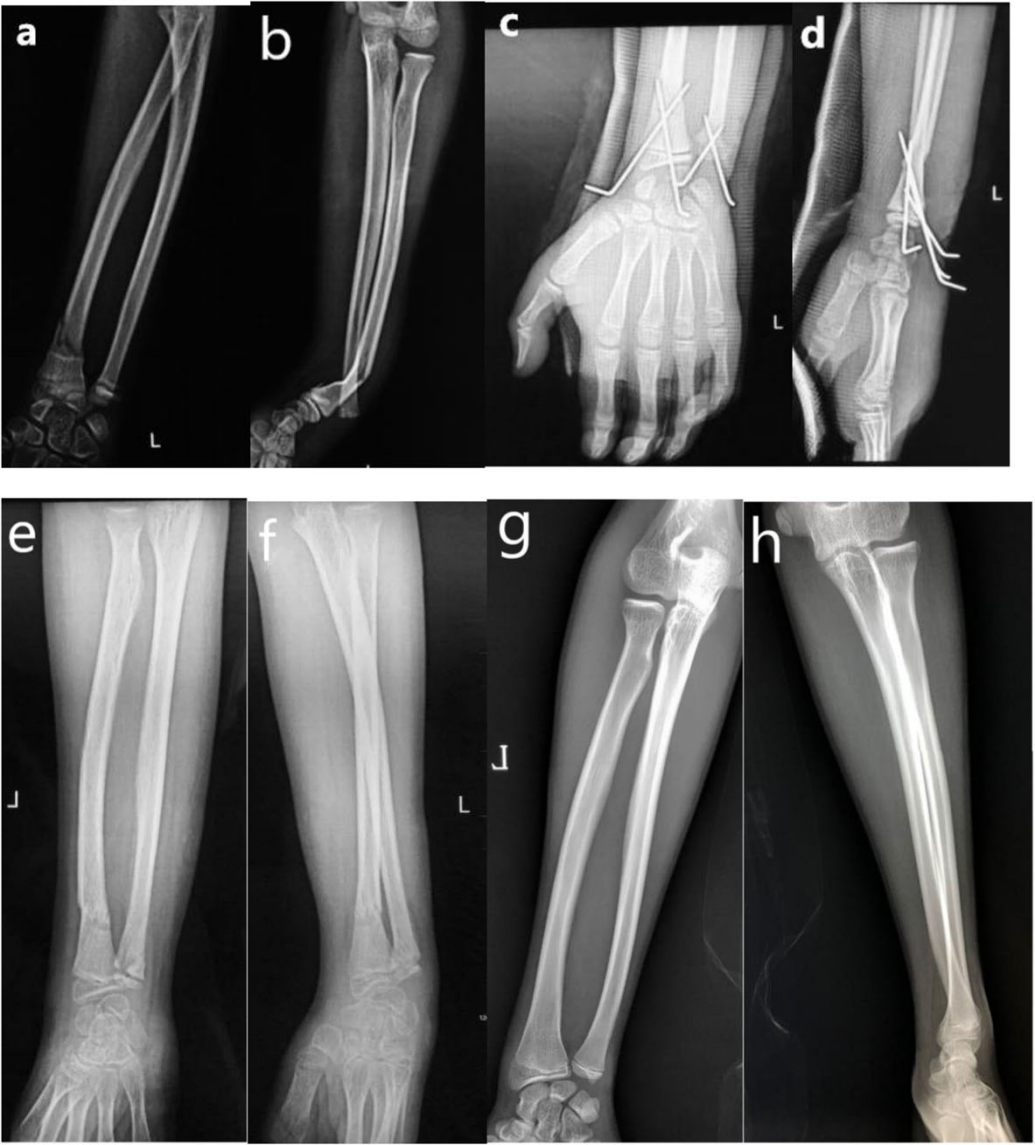
13-year-old boy with left distal radius-ulna fracture treated with crossed KW fixation. **a** AP view of forearm before surgery. **b** Lateral view of forearm before surgery. **c** AP view of wrist joint after surgery. **d** Lateral view of wrist joint after surgery. **e** AP view of forearm after Kirschner wire removal. **f** Lateral view of forearm after Kirschner wire removal. **g** AP view of forearm at 18th month follow-up. **h** Lateral view of forearm at 18th month follow-up

**Fig. 4:**
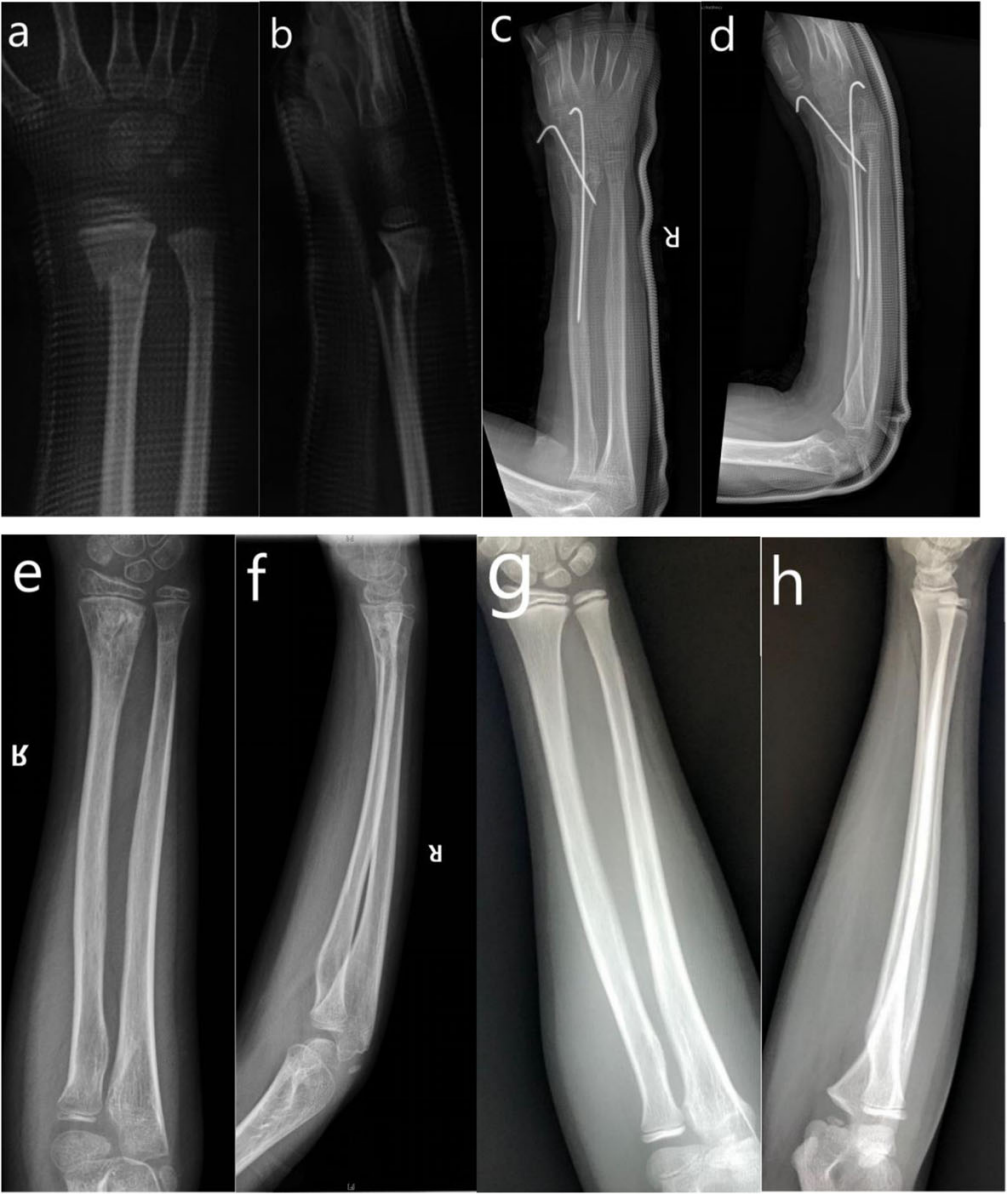
11-year-old boy of right distal radius-ulna fracture treated with crossed KW fixation. **a**. AP view of wrist joint before surgery. **b**. Lateral view of wrist joint before surgery. **c**. AP view of full-length forearm after surgery. **d**. Lateral view of full-length forearm after surgery. **e**. AP view of forearm at 9th month follow-up. **f**. Lateral view of forearm at 9th month follow-up. **g**. AP view of forearm at 15th month follow-up. **h**. Lateral view of forearm at 15th month follow-up

Crossed K-wire fixation was performed as follows. Under fluoroscopy guidance, the fracture was manually reduced, and a pair of K-wires were inserted in a crossed fashion to fixate the fracture. If manual reduction failed, a pin was used as a lever to facilitate reduction (See Fig. 3 and Fig. 4).

After surgery, all patients were immobilized in short or long arm cast for 3–4 weeks. The EF was usually removed at 4–8 weeks at the out-patient department, as per the clinical and radiographic evidence of fracture union. The KWs were removed similarly as EF. After the removal of hardware, a short-arm brace was used for the patient for additional 3–4 weeks.

### Follow-up

Patients were followed-up for at least 12 months after index surgery. Data, including the time of hardware removal, range of forearm rotation, range of wrist motion, fracture re-displacement and postoperative complications, were collected and recorded.

### Statistical analysis

SPSS statistical package program (SPSS 19.0 version; SPSS Inc., Chicago, Illinois, USA) was used for statistical analysis. Data were presented as mean ± SD (range), median (range), or n (%). χ2-test (categorical data) or Student’s t-test (continuous data) was used to compare the results from two groups. And a *P*-value of <.05 was considered significantly different.

## Results

In all, 146 patients (male 101, female 45) in the EF and 117 patients (male 76, female 41) in the KW group were included, and all the patients were followed up for more than 12 months. As shown in Table 1, there existed no statistically significant differences between the two groups regarding sex, body weight, fracture side, duration from injury to surgery. The duration of surgery was significantly shorter in the EF (30.5 ± 6.1 min) than the KW group (44.6 ± 9.4 min) (*P* < 0.001). The number of intraoperative X-ray images was significantly lower in the EF (6.5 ± 1.1) than the KW (11.8 ± 2.3), (*P* < 0.001).

**Table 1:** Demographic and clinical parameters of children with radial MDJ fractures in EF and KW group

Parameters	EF (*n* = 146)	KW(*n* = 117)	*P* Value
Age, years	12.1 ± 1.4	11.9 ± 1.5	0.322
Sex, male/female	101/45	76/41	0.469
Body Weight (kg)	33.6 ± 4.2	33.9 ± 4.9	0.583
Fracture side, L/R	84/62	74/43	0.347
Combined ulnar fracture (%)	20.5	19.7	0.858
Duration of surgery, min	30.5 ± 6.1	44.6 ± 9.4	< 0.001^*^
Images taken during the operation	6.5 ± 1.1	11.8 ± 2.3	< 0.001^*^
From injury to surgery (d)	5.1 ± 1.4	5.3 ± 1.4	0.266

Data shown as mean ± SD or N(%)

* < 0.01

As shown in Table 2, there existed no statistically significant differences between the EF and KW group regarding the time from surgery to implant removal, pin tract infection, loss of forearm rotation, range of wrist motion. The incidence of tendon irritation is significantly higher in the KW (19.7%) than the EF group (0%) (*P* < 0.001). The residual angulation on the AP view was higher in the KW (3.8 ± 2.3 degrees) than the EF group (2.5 ± 1.6, degrees), *P* < 0.001. The volar tilting is better in the EF (6.6 ± 1.1, degrees) than the KW group (1.0 ± 1.5 degrees) (*P* < 0.001).

**Table 2:** Follow-up data for children with radial MDJ fractures in EF and KW group

Parameter	EF (n = 146)	KW (n = 117)	P Value
Hardware removal, weeks	6.5 ± 1.1	6.6 ± 1.1	0.492
Pin tract infection (%)	10.3	10.3	0.997
Tendon Irritation (%)	0	19.7	< 0.001^*^
Forearm pronation (°)	87.2 ± 1.6	87.2 ± 1.6	0.932
Forearm supination (°)	87.6 ± 1.7	87.7 ± 1.7	0.596
DASH score	12.3 ± 1.7	12.7 ± 1.7	0.086
Angulation(°)	2.5 ± 1.6	3.8 ± 2.3	< 0.001^*^
Volar tilt(°)	6.6 ± 1.1	1.0 ± 1.5	< 0.001^*^

Data shown as mean ± SD or N(%)

Angulation = angulation on frontal plane

* < 0.01

As shown in Table 3, 30 patients (male 20, female 10) in the EF and 23 patients (male 15, female 8) in the KW group presented with both-bone fractures of the forearm. There existed no significant difference between the two groups regarding sex, body weight, fracture side, duration from injury to surgery. The duration of surgery was significantly shorter in the EF (31.9 ± 5.9, min) than the KW group (44.2 ± 9.6, min) (*P* < 0.001). The number of intraoperative X-ray images was significantly lower in the EF (6.6 ± 1.1) than KW (12.4 ± 2.4) (*P* < 0.001).

**Table 3:** Demographic and clinical parameters of children with distal forearm both-bone fractures

Parameters	EF (*n* = 30)	KW (*n* = 23)	P Value
Age, years	11.7 ± 1.3	12.2 ± 1.6	0.187
Sex, male/female	20/10	15/8	0.907
Body Weight (kg)	33.1 ± 3.9	35.1 ± 4.8	0.111
Fracture side, L/R	20/10	14/9	0.644
Duration of surgery, min	31.9 ± 5.9	44.2 ± 9.6	< 0.001^*^
Images taken during the operation	6.6 ± 1.1	12.4 ± 2.4	< 0.001^*^
From injury to surgery (d)	5.6 ± 1.3	5.3 ± 1.2	0.460

*EF* External fixator, *KW* Kirschner wire

Data shown as mean ± SD or N(%)

* < 0.01

As shown in Table 4, for patients with both-bone fractures of the forearm, there existed no significant difference between EF and KW group regarding the time from surgery to implant removal, pin tract infection, loss of forearm rotation, range of wrist motion. The incidence of tendon irritation is significantly higher in the KW group (26.1%) than the EF group (0%), *P* < 0.001. The residual angulation on the AP view was higher in KW (4.2 ± 2.5, degrees) than the EF group (2.7 ± 1.4, degrees), *P* = 0.018. The volar tilting is better in the EF (6.5 ± 1.1, degrees) than the KW group (1.3 ± 1.3, degrees) (*P* < 0.001).

**Table 4:** Follow-up data for children with distal forearm both bone fractures in EF and KW group

Parameter	EF (n = 30)	KW (n = 23)	P Value
Hardware removal, weeks	6.4 ± 1.1	6.6 ± 0.9	0.463
Pin tract infection (%)	10.0	4.3	0.462
Tendon Irritation (%)	0	26.1	< 0.001^*^
Forearm pronation (°)	87.4 ± 1.5	87.3 ± 1.5	0.680
Forearm supination (°)	87.5 ± 1.6	86.7 ± 1.6	0.092
DASH score	12.4 ± 1.5	12.7 ± 1.4	0.517
Angulation(°)	2.7 ± 1.4	4.2 ± 2.5	0.018
Volar tilt(°)	6.5 ± 1.1	1.3 ± 1.3	< 0.001^*^

Data shown as mean ± SD or N(%)

Angulation = angulation on frontal plane

* < 0.01

As shown in Table 5, 116 patients (male 81, female 35) in the EF and 94 patients (male 61, female 33) in the KW group presented with isolated radial MDJ fractures. There existed no significant difference between the two groups regarding sex, body weight, fracture side, duration from injury to surgery. The duration of surgery was significantly shorter in the EF (30.2 ± 6.2, min) than the KW group (44.7 ± 9.4, min) (*P* < 0.001). The number of intraoperative X-ray images was significantly lower in the EF (6.5 ± 1.1) than KW (11.6 ± 2.2) (*P* < 0.001).

**Table 5:** Demographic and clinical parameters of children with isolated radial MDJ fractures in EF and KW group

Parameters	EF (*n* = 116)	KW (*n* = 94)	P Value
Age, years	12.2 ± 1.4	11.9 ± 1.5	0.074
Sex, male/female	81/35	61/33	0.441
Body Weight (kg)	33.7 ± 4.3	33.6 ± 4.8	0.859
Fracture side, L/R	64/52	60/34	0.204
Duration of surgery, min	30.2 ± 6.2	44.7 ± 9.4	< 0.001^*^
Images taken during the operation	6.5 ± 1.1	11.6 ± 2.2	< 0.001^*^
From injury to surgery (d)	5.0 ± 1.4	5.3 ± 1.4	0.114

*EF* External fixator, *KW* Kirschner wire

Data shown as mean ± SD or N(%)

* < 0.01

As shown in Table 6, for patients with both-bone fractures of the forearm, there existed no significant differences between the EF and KW group regarding the time from surgery to implant removal, pin tract infection, loss of forearm rotation, range of wrist motion. The incidence of tendon irritation is significantly higher in the KW group (18.1%) than the EF group (0%) (*P* < 0.001). The residual angulation on the AP view was higher in the KW (3.6 ± 2.2 degrees) than the EF group (2.5 ± 1.7 degrees) (*P* < 0.001). The volar tilting is better in the EF (6.7 ± 1.1 degrees) than the KW group (0.9 ± 1.5 degrees) (*P* < 0.001).

**Table 6:** Follow-up data for children with isolated radial MDJ fracture in EF and KW group

Parameter	EF (n = 116)	KW (n = 94)	P Value
Hardware removal, weeks	6.5 ± 1.1	6.6 ± 1.2	0.677
Pin tract infection (%)	10.3	11.7	0.756
Tendon Irritation (%)	0	18.1	< 0.001^*^
Forearm pronation (°)	87.2 ± 1.7	87.2 ± 1.7	0.991
Forearm supination (°)	87.6 ± 1.7	87.9 ± 1.7	0.155
DASH score	12.3 ± 1.7	12.7 ± 1.7	0.112
Angulation(°)	2.5 ± 1.7	3.6 ± 2.2	< 0.001^*^
Volar tilt(°)	6.7 ± 1.1	0.9 ± 1.5	< 0.001^*^

Data shown as mean ± SD or N(%)

Angulation = angulation on frontal plane

* < 0.01

No patient in the EF and KW groups required re-operation. Five patients in the EF and 4 patients in the KW suffered refracture after hardware removal. Three patients in the EF resulted from an accidental fall onto the ground, and 2 resulted from accidental bumping into the hard surface. Four patients in the KW group results from accidental fall.

## Discussion

The most important finding of our study was that radiograph showed better volar tilting and less angulation in the EF than KW. Besides, EF displayed shorter duration of surgery, less tendon irritation than the KW. Therefore, the EF was superior to KW in the treatment of delayed MDJ fractures in adolescents.

Some authors advocated the use of EF in the shaft of radius fractures in children [11, 12], and some reported the use of non-bridging EF for the distal radius fractures in adults [13–16]. Even recently, Korobeinikov et al. reported the utilization of the Ilizarov frame for the distal radius fracture in children, with satisfactory clinical outcomes [17]. However, none of the previous studies reported the utilization of simple EF for the treatment of MDJ fractures in children. Generally, there are three types of EF available for adults [18]. These include F-wrist, Hoffman II Compact, and Pennig Dynamic Wrist Fixator, but such devices are not available in our hospital. Hybrid EF is readily available and relatively easy to assemble, which utilizes the different sizes of Schanz screws and clamps. Though it looks bulky, patient compliance is good.

Displaced distal radial fractures (DDRF) in children and adolescents usually require intervention that varies from simple manual reduction followed by well-molded casting to KW fixation and even plating [19]. In children with DDRF, the re-operation rate following manipulation under anesthesia alone ranges from 14 to 91% [20]. The limited remodeling potential and high demand for wrist function among teenagers require an acceptable reduction and adequate stabilization. Most authors prefer additional KW fixation over casting alone [21, 22]. Lieber et al. even reported the use of transepiphyseal intramedullary KW for the treatment of unstable MDJ fractures in children [4]. However, the iatrogenic damage to physis cannot be overlooked, and if the KW diameter is too small, the stability of reduction is questionable. Therefore, the authors recommended additional stabilization.

In our study, the operative time was shorter in the EF group than the KW group. In order to provide reliable stability, the KW advancement should remain at an acute angle between the KW trajectory and the skin. However, cortical penetration is quite challenging in this technique. Kapandji technique has been reported in the treatment of DDRF in children [23], but it is not the mainstream choice and not adopted in our institute. In clinical practice, multiple KWs placement might be required for an adequate reduction and stable construct; however, it might increase the risk of iatrogenic injury [24]. In contrast, the threaded screw in the EF provides a “joystick” and is conducive to reduction. The placement of the Schanz screw under fluoroscopy is also relatively simple.

The purpose of surgical intervention is to reduce the risk of redisplacement following fracture reduction. The redisplacement in both the groups was considerably low, and no patient in our study required re-operation. The EF group showed better radiographic results in angulation on AP view and volar tilting, possibly due to better stability in EF than KW.

Our patients were divided into two subgroups as per the presence or absence of the ulna fracture. In patients with both-bone fractures of the forearm, the operative time was relatively longer. However, ulna was not routinely fixated in our case series; some surgeon still prefers to fixate the ulna for better stability.

A long-arm or short-arm slab was routinely applied after the surgery for 3–4 weeks. After the radiograph showing the evidence of callus formation, the slab was removed. However, if the patient was not compliant, prolonged use of cast was mandatory. After the removal of hardware, a short-arm brace was used for 3–4 weeks for every patient to reduce the risk of refracture, which might be the reason for the low rate of refracture in our study. Besides, sports activities were not allowed until full consolidation of the fracture on the radiographs.

Pin tract infection (PTI) was reported to be a common complication in the EF and KW [25, 26], however, all PTI resolved uneventfully with oral antibiotics. The incidence of tendon irritation was higher in the KW because the procedure was performed in percutaneous fashion, whereas the EF was performed using a small incision exposing the bone.

Despite an adequate sample size, several limitations exist in this study. Firstly, other fixation techniques, including ESIN and plate, were not included. Secondly, the number of X-rays exposure was recorded, yet the actual radiation dosage could not be meticulously measured and recorded. Thirdly, the cost-effect analysis was not performed in this study.

## Conclusion

The EF was superior to KW in the treatment of radial MDJ fractures in adolescents.

The EF displayed shorter duration of surgery, less tendon irritation, and better radiographic outcomes than the KW. However, the cost-effect analysis remains to be investigated, because the EF is more expensive than KW.

## Data Availability

The data sets supporting the conclusion of this article are included within the article. Upon request, raw data can be provided by the corresponding author.

## Abbreviations

KW: Kirschner-wiring
MDJ: Metaphyseal diaphyseal junction
EF: External fixator
